# Supplementary material for: Tailoring transcranial alternating current stimulation based on endogenous event-related P3 to modulate premature responses: a feasibility study
Source: PeerJ. 2024 Apr 3;12:e17144. doi: 10.7717/peerj.17144 (PMC10998630; doi:10.7717/peerj.17144)
Supplement: Supplemental Information 1 [file peerj-12-17144-s001.docx]

***Supplementary Materials***

Tailoring transcranial alternating current stimulation based on endogenous event related P3 to modulate premature responses: a feasibility study

**S.M. 1. Self-report Questionnaires**

***S.M. 1.1.* *Sociodemographic, impulsivity, and clinical information***

**Table S1.** Sociodemographic characteristics, impulsivity measures and clinical symptomatology per participant.

|  | | Sex | | Age | | EHI | BIS | S-UPPS | DASS | | |  |
| --- | --- | --- | --- | --- | --- | --- | --- | --- | --- | --- | --- | --- |
|  |  |  |  |  |  |  |  |  | Depression | Anxiety | Stress | |
| Participant 1 | F | | 25 | | 100 | | 49 | 35 | 4 | 4 | 8 | |
| Participant 2 | M | | 28 | | 100 | | 52 | 33 | 0 | 4 | 6 | |
| Participant 3 | M | | 24 | | 100 | | 58 | 51 | 8 | 0 | 0 | |
| Participant 4 | M | | 26 | | 100 | | 58 | 50 | 8 | 0 | 2 | |
| Participant 5 | M | | 22 | | 100 | | 69 | 48 | 0 | 0 | 0 | |
| Participant 6 | F | | 26 | | 100 | | 51 | 33 | 0 | 0 | 0 | |
| Participant 7 | F | | 29 | | 66.67 | | 53 | 39 | 2 | 0 | 8 | |
| Participant 8 | F | | 27 | | 80 | | 58 | 40 | 2 | 0 | 4 | |
| Participant 9 | F | | 27 | | 100 | | 48 | 25 | 0 | 0 | 0 | |
| Participant 10 | M | | 24 | | 89.47 | | 62 | 30 | 0 | 4 | 2 | |
| Participant 11 | F | | 26 | | 57.14 | | 54 | 36 | 10 | 6 | 12 | |
| Participant 12 | F | | 24 | | 86.67 | | 56 | 36 | 4 | 4 | 10 | |

***S.M. 1.2.* *Blinding***

| **Participant** | **Active Session** | | **Sham Session** | |
| --- | --- | --- | --- | --- |
|  | **Guess** | **Confidence** | **Guess** | **Confidence** |
| 1  **Table S2.** Results of the tACS blinding questionnaire per participant. | Active | 4 | Active | 3 |
| 2 | Active | 2 | Placebo | 3 |
| 3 | Placebo | 5 | Placebo | 1 |
| 4 | Placebo | 2 | Active | 3 |
| 5 | Placebo | 3 | Active | 4 |
| 6 | Placebo | 2 | Placebo | 3 |
| 7 | Placebo | 1 | Active | 0 |
| 8 | Active | 2 | Placebo | 1 |
| 9 | Placebo | 4 | Active | 3 |
| 10 | Active | 3 | Placebo | 3 |
| 11 | Placebo | 2 | Active | 2 |
| 12 | Placebo | 1 | Placebo | 4 |
| Correct Guess | 28.6 |  | 42.9 |  |
| Wrong Guess | 71.4 |  | 57.1 |  |

**Legend:** 0 – “No confident at all”; 1 – “Slightly confident”; 2 – “Moderately confident”; 3 – “Considerably confident”; 4 – “Extremely confident”

**S.M. 2. Examples of synchronization between the peak of tACS and the target-P3 latency**


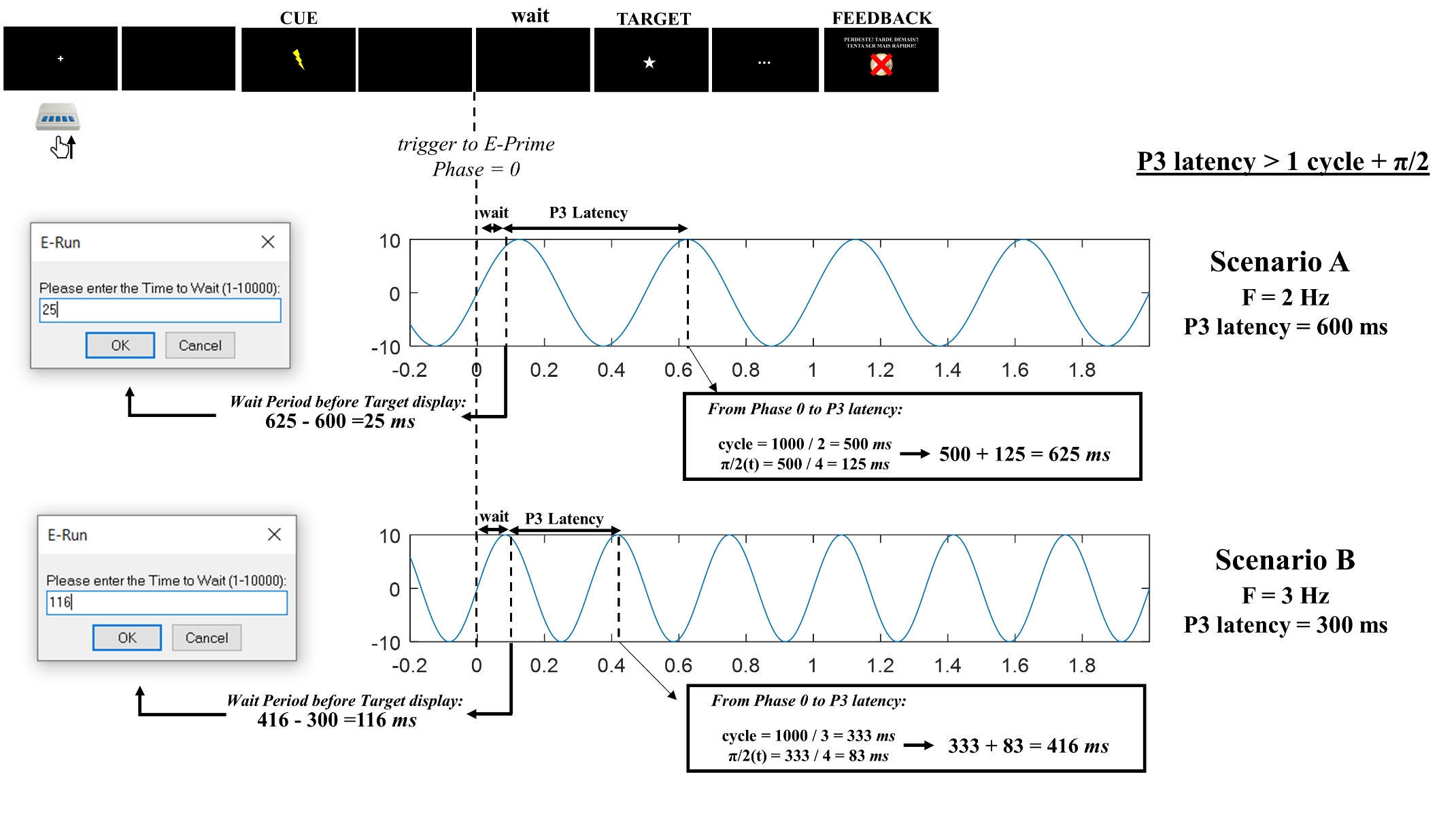


**Figure S1.** Examples of synchronization between the tACS peak and P3 latency in CPRT using E-Prime 3. These scenarios correspond to examples that P3 latency is higher than the time of 1 cycle + π/2 from tACS frequency.

**Figure S2.** Examples of synchronization between the tACS peak and P3 latency in CPRT using E-Prime 3. These scenarios correspond to examples that P3 latency is lower than the time of 1 cycle + π/2 from tACS frequency, which requires 2 cycles + π/2 (Scenario C) or 3 cycles + π/2 (Scenario D) to achieve the match.


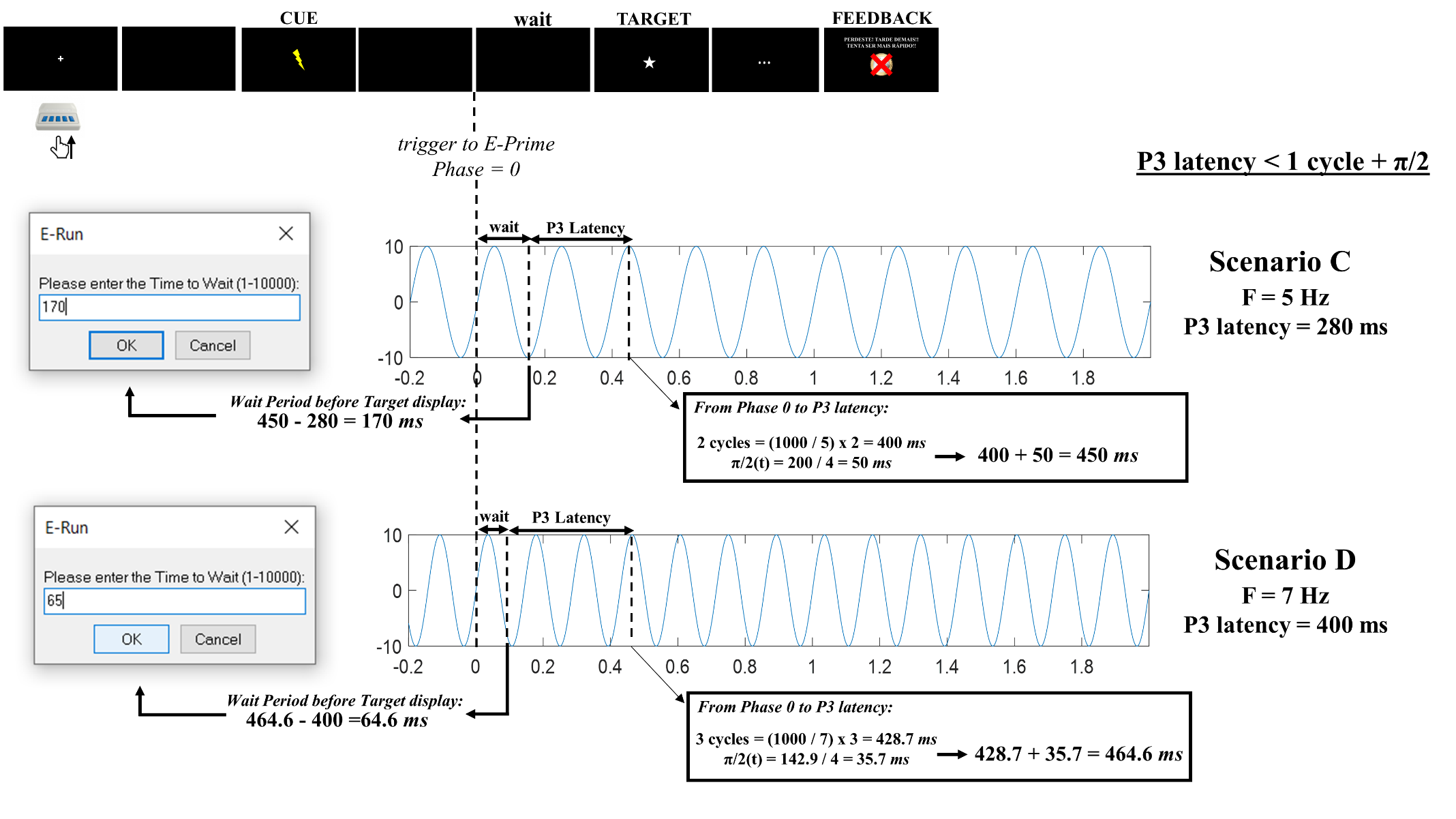


**S.M. 3. EEG online analysis results and parameters of reward/punishment system**

**Table S3.**  Online P3 results for subsequent tACS-EEG synchronization and the average/SD RT of the training block.

|  | | tACS frequency (Hz) | | P3 latency (ms) | | “Wait” period (ms) | | Mean RT in Training trials (ms) | | SD RT in Training trials (ms) | |
| --- | --- | --- | --- | --- | --- | --- | --- | --- | --- | --- | --- |
| Participant 1 | 7 | | 280 | | 152.3 | | 200.14 | | 113.8 | |  |
| Participant 2 | 6 | | 292 | | 217.7 | | 155.6 | | 51.1 | |  |
| Participant 3 | 2.25 | | 352 | | 203.6 | | 391.4 | | 127.6 | |  |
| Participant 4 | 2.25 | | 302 | | 253.6 | | 156.9 | | 62.4 | |  |
| Participant 5 | 1.75 | | 312 | | 402.3 | | 209.5 | | 109.2 | |  |
| Participant 6 | 2.5 | | 290 | | 210 | | 210.9 | | 57 | |  |
| Participant 7 | 3 | | 364 | | 52.7 | | 294.1 | | 72.1 | |  |
| Participant 8 | 2 | | 364 | | 261 | | 160.2 | | 57.4 | |  |
| Participant 9 | 1.5 | | 302 | | 531.3 | | 288.1 | | 90.6 | |  |
| Participant 10 | 3 | | 300 | | 116.7 | | 157.1 | | 55.7 | |  |
| Participant 11 | 2.25 | | 368 | | 187.6 | | 211.9 | | 58.9 | |  |
| Participant 12 | 6 | | 290 | | 251.7 | | 193.2 | | 35.9 | |  |

**S.M. 4. Reinforcement/punishment feedback in CPRT**


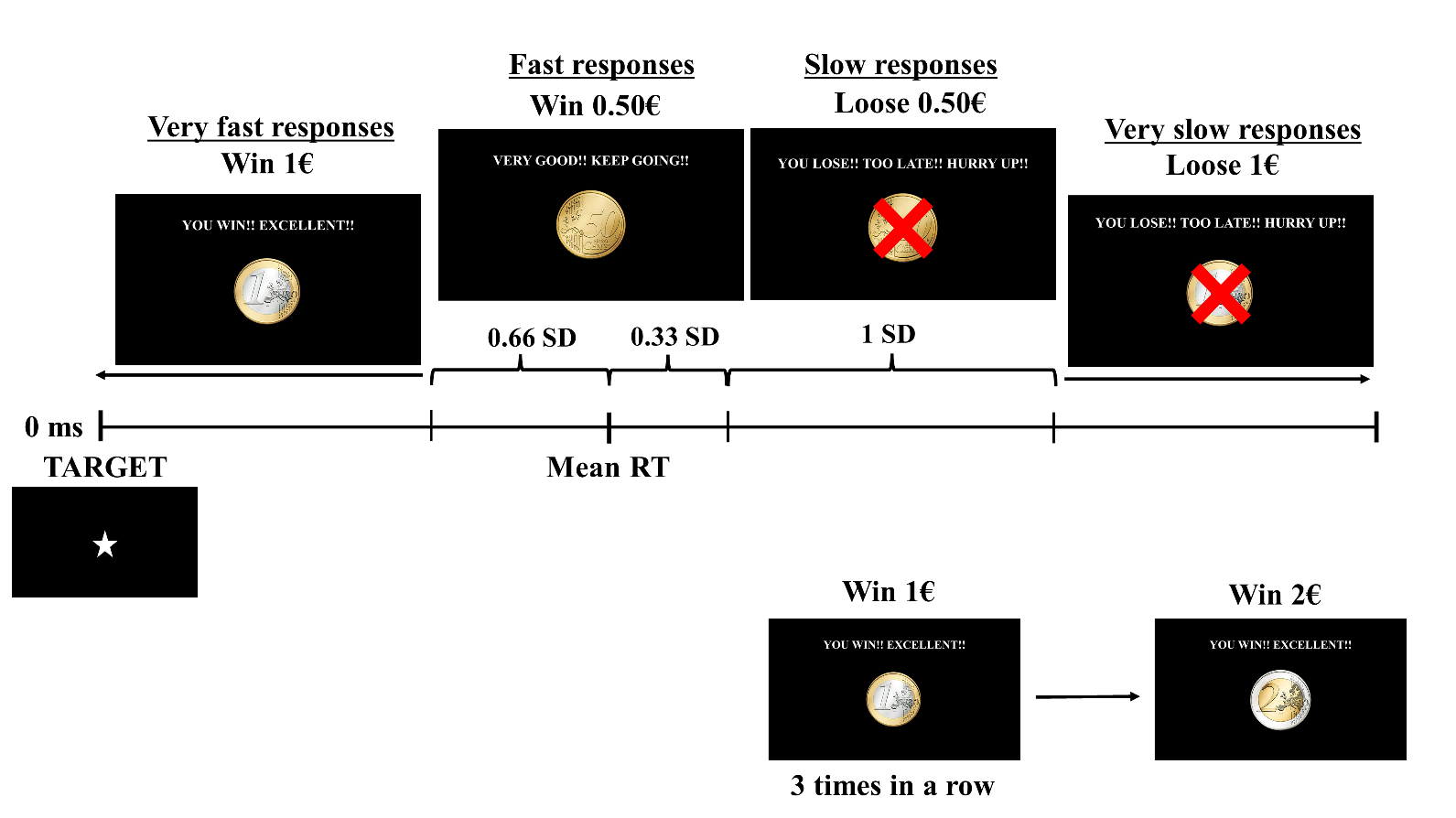


**Figure S3.** The tailored reward/punishment system from the CPRT. The feedback was dependent on the mean and SD of the release time from the last 10 trials of the baseline block.

**S.M. 5. Examples of time and frequency windows in the additional analysis**

**Table S4.** Total number of epochs in the different steps of the preprocessing of EEG files.

|  |  | Target-P3 | | | |  | Cue-P3 | | | | | |
| --- | --- | --- | --- | --- | --- | --- | --- | --- | --- | --- | --- | --- |
|  |  | Initial epochs | 100 µV | Visual inspection | Rejection Rate (%) |  | Initial epochs | Premature response | 800ms cue-target interval | 100 µV | Visual inspection | Rejection Rate (%) * |
| Active | Pre | 82.82 | 80.36 | 75.36 | 9.01 |  | 97.36 | 95.64 | 83 | 79.46 | 72.18 | 13.04 |
|  | Post | 83.91 | 82.27 | 76.09 | 9.32 |  | 98 | 95.09 | 81.27 | 80.81 | 74.09 | 8.83 |
| Sham | Pre | 75.27 | 74.63 | 70.82 | 5.91 |  | 88 | 86.18 | 74.73 | 74 | 58.82 | 21.29 |
|  | Post | 83.09 | 82.82 | 79.09 | 4.81 |  | 96.82 | 94.73 | 81.36 | 81.27 | 74.27 | 8.71 |

* Rejection rate comprising the number of epochs removed exceeding the ±100 µV and visual inspection

**
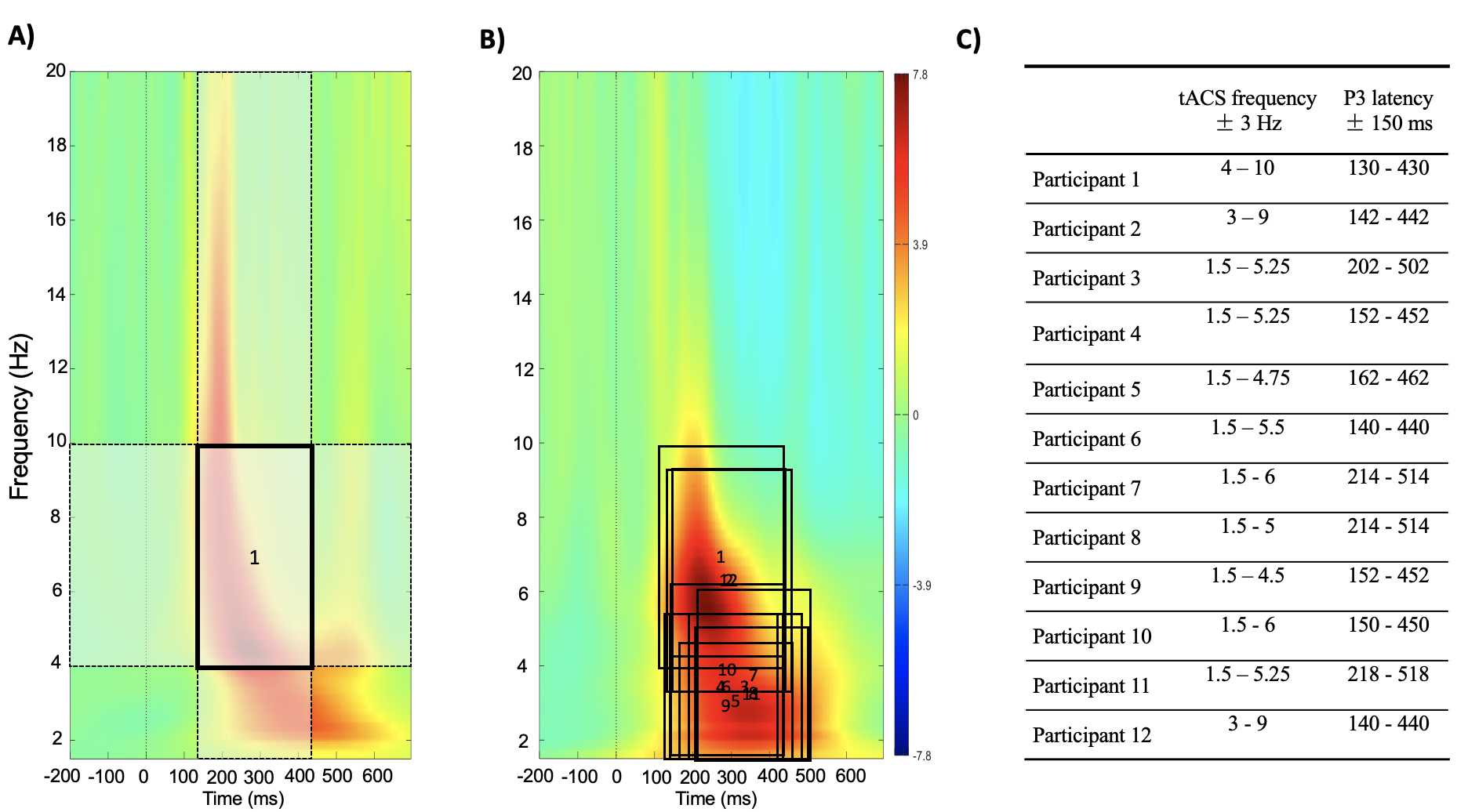
S.M. 6. Examples of time and frequency windows in the additional analysis**

**Figure S4.** The additional analysis extracted the ERO power from frequency and temporal windows according to the tACS frequency (± 3 Hz) and the P3 latency (± 150 ms). The first figure (A) represents both windows considering the frequency (4 – 10 Hz) and temporal window (130 – 430 ms) of Participant 1. The second figure (B) represents all the windows used to extract ERO from the 12 participants and the table (C) comprises all the values of the windows.

**S.M. 7. P3 latency during the experimental blocks of CPRT**

**Table S5.** P3 latency during baseline, pre- and post-stimulation in both sessions and corresponding difference.

|  |  |  |  |  |  |  |  |  |  |  |
| --- | --- | --- | --- | --- | --- | --- | --- | --- | --- | --- |
| Subject | P3 latency | | | | |  | Difference between P3 latency | | | |
|  | Baseline | Pre-Active | Post-Active | Pre-Sham | Post-Active |  | Baseline - Pre-Active | Baseline - Post-Active | Baseline - Pre-Sham | Baseline - Post-Sham |
| 1 | 280 | 284 | 284 | 300 | 340 |  | 4 | 4 | 20 | 60 |
| 2 | 292 | 298 | 412 | 300 | 300 |  | 6 | 120 | 8 | 8 |
| 3 | 352 | 274 | 380 | 416 | 308 |  | -78 | 28 | 64 | -44 |
| 4 | 302 | 302 | 306 | 286 | 432 |  | 0 | 4 | -16 | 130 |
| 5 | 312 | 312 | 316 | 328 | 326 |  | 0 | 4 | 16 | 14 |
| 6 | 290 | 252 | 252 | 302 | 274 |  | -38 | -38 | 12 | -16 |
| 7 | 364 | 358 | 338 | 358 | 334 |  | -6 | -26 | -6 | -30 |
| 8 | 364 | 422 | 586 | 366 | 536 |  | 58 | 222 | 2 | 172 |
| 9 | 302 | 322 | 334 | 292 | 322 |  | 20 | 32 | -10 | 20 |
| 10 | 300 | 316 | 326 | 314 | 442 |  | 16 | 26 | 14 | 142 |
| 11 | 368 | 320 | 306 | 298 | 368 |  | -48 | -62 | -70 | 0 |
|  |  |  |  |  |  |  |  |  |  |  |
