# Supplementary material for: Tailoring transcranial alternating current stimulation based on endogenous event-related P3 to modulate premature responses: a feasibility study
Source: PeerJ. 2024 Apr 3;12:e17144. doi: 10.7717/peerj.17144 (PMC10998630; doi:10.7717/peerj.17144)
Supplement: Supplemental Information 2 — Original version of the blinding questionnaire used [file peerj-12-17144-s002.pdf]

Iniciais do sujeito: ______ Protocolo #_______________

Data: ___/___/___

Nome do(s) investigador(es): ____

Título do estudo (abreviado): ____

Validação do Blinding

Por favor, indique se é o investigador, o avaliador ou o participante:

-Investigador (__)

-Avaliador (__)

-Participante (__)

Por favor, indique o seu melhor palpite, sobre qual o grupo de estimulação em que foi inserido:

-Experimental (__)

-Placebo (__)

-Não sei (__)

Quão confiante está de que o seu palpite está correto?

- Extremamente (__)

-Consideravelmente (__)

-Moderadamente (__)

-Levemente (__)

-De modo nenhum (__)
