# Supplementary material for: Tailoring transcranial alternating current stimulation based on endogenous event-related P3 to modulate premature responses: a feasibility study
Source: PeerJ. 2024 Apr 3;12:e17144. doi: 10.7717/peerj.17144 (PMC10998630; doi:10.7717/peerj.17144)
Supplement: Supplemental Information 3 — Blinding questionnaire in English [file peerj-12-17144-s003.pdf]

Subject: ______ Protocol #_______________

Date: ___/___/___

Researcher name: ____

Title of the study: ____

Blinding

Please indicate whether you are the researcher, reviewer or participant:

-Researcher(__)

-Rater (__)

-Participant (__)

Please indicate your best guess as to which stimulation group you were placed in:

-Active (__)

-Sham (__)

-I don’t know (__)

How confident are you that your guess is correct?

- Extremely (__)

-Considerably (__)

-Moderate (__)

-Slightly (__)

-Not at all (__)
